# Cooperative partner choice in multi-level male dolphin alliances

**DOI:** 10.1038/s41598-021-85583-x

**Published:** 2021-03-25

**Authors:** Livia Gerber, Samuel Wittwer, Simon J. Allen, Kathryn G. Holmes, Stephanie L. King, William B. Sherwin, Sonja Wild, Erik P. Willems, Richard C. Connor, Michael Krützen

**Affiliations:** 1 Evolutionary Genetics Group, Department of Anthropology, University of Zurich, 8057 Zurich, Switzerland; 2 School of Biological Sciences, University of Bristol, Bristol, BS8 1TQ UK; 3 School of Biological Sciences and Oceans Institute, University of Western Australia, Crawley, WA 6009 Australia; 4 Evolution and Ecology Research Centre, School of Biological, Earth and Environmental Sciences, UNSW Sydney, Sydney, NSW 2052 Australia; 5 Centre for the Advanced Study of Collective Behaviour, University of Konstanz, 78464 Konstanz, Germany; 6 Cognitive and Cultural Ecology Research Group, Max Planck Institute of Animal Behavior, 78315 Radolfzell, Germany; 7 Biology Department, UMASS Dartmouth, North Dartmouth, MA 02747 USA

**Keywords:** Behavioural ecology, Animal behaviour, Evolutionary biology, Evolutionary genetics, Social evolution

## Abstract

Investigations into cooperative partner choice should consider both potential and realised partners, allowing for the comparison of traits across all those available. Male bottlenose dolphins form persisting multi-level alliances. Second-order alliances of 4–14 males are the core social unit, within which 2–3 males form first-order alliances to sequester females during consortships. We compared social bond strength, relatedness and age similarity of potential and realised partners of individual males in two age periods: (i) adolescence, when second-order alliances are formed from all available associates, and (ii) adulthood, when first-order allies are selected from within second-order alliances. Social bond strength during adolescence predicted second-order alliance membership in adulthood. Moreover, males preferred same-aged or older males as second-order allies. Within second-order alliances, non-mating season social bond strength predicted first-order partner preferences during mating season consortships. Relatedness did not influence partner choice on either alliance level. There is thus a striking resemblance between male dolphins, chimpanzees and humans, where closely bonded non-relatives engage in higher-level, polyadic cooperative acts. To that end, our study extends the scope of taxa in which social bonds rather than kinship explain cooperation, providing the first evidence that such traits might have evolved independently in marine and terrestrial realms.

## Introduction

Competition and cooperation are inherent to all forms of life, found in cellular mechanisms through to the formation and maintenance of complex societies^1^. Both influence access to vital resources such as food and mates, with the underlying mechanisms for competition explained by Darwin via natural selection and the ‘struggle for existence’^2^. However, we still lack a complete understanding of some of the underlying proximate mechanisms of cooperation, including who cooperates with whom and when^3–6^. Such cooperative partner choice is particularly interesting when individuals have many partners from whom to choose, as is the case in large, complex societies that involve a high degree of social mixing and well-differentiated relationships.

Kinship frequently plays a role in driving the propensity for cooperation between individuals^7–10^. Relatives share genes by descent, which allows individuals to gain indirect fitness benefits when cooperating with kin^11^. Yet, in settings where the outcome of cooperative acts depends on certain attributes of a partner (e.g., strength in a fight), individuals may accrue most fitness benefits when choosing the most competent individual rather than the closest relative^12^. Partner choice based on competence requires that individuals identify and are able to recruit the most valuable partners. To achieve this, individuals can either bid for valuable partners by offering services, grooming for example, and in exchange, gain benefits such as coalitionary support^13^. Alternatively, individuals may selectively invest in relationships with potentially valuable conspecifics, resulting in the formation of social bonds, here defined as persisting affiliative relationships among individuals^14,15^. Social bonds often facilitate cooperation, at least in long-lived species^16,17^ e.g., zebra finches (*Taeniopygia guttata*)^18^, Barbary macaques (*Macaca sylvanus*)^19^, reviewed in Refs.^20,21^. Complex social relationships, during which individuals selectively invest in particular partners, occur primarily in species with low levels of average relatedness^22^.

Although kin are preferred as social partners in many societies, even where relatives are scarce, this is not always the case across species or contexts^23^. In greater anis (*Crotophaga major*), for example, unrelated individuals nest cooperatively to increase their reproductive success^24^, while bats (*Desmodus rotundus*) feeding kin as well as non-kin benefit from larger food donations compared to those only feeding their relatives^25^. Similarly, in complex tasks requiring competent partners, cooperative partner choice is not necessarily based on relatedness. In chimpanzees (*Pan troglodytes*), unrelated but closely bonded males participate in border patrols, share meat after hunts and form competitive coalitions^26–28^.

Male alliances are particularly interesting in the context of partner choice, since they must cooperate, rather than compete, to gain access to females, yet fertilisations cannot be shared^29^. Alliance formation in bottlenose dolphins (*Tursiops* spp.) is intriguing because kin selection appears to explain partner choice in some populations^30,31^ but not in others^32,33^, while the complexity of alliances vary^34^. Cooperation in this species may therefore reveal mechanisms other than kin selection, such as various forms of reciprocity^35,36^ or by-product mutualism^37,38^. Male Indo-Pacific bottlenose dolphins (*T. aduncus*, ‘dolphins’ hereafter) in Shark Bay form nested alliances in an unbounded social network with high fission–fusion dynamics^39^. Alliance membership is pivotal for male fitness, since non-allied males father no or very few offspring^40^. Second-order alliances, the core male social unit, can last for decades and comprise up to 14 adult males, within which two to three males cooperate in first-order alliances to sequester single oestrus females in events known as consortships^41^. First-order alliances vary in composition and stability, with many males showing clear preferences for particular individuals when forming first-order alliances within their second-order alliances^41,42^. Attacks from other alliances in attempts to steal a consorted female are defended on both alliance levels^41^. Allied males associate throughout the year, despite the fact that mating is markedly seasonal^43,44^.

To date, the effect of relatedness on male alliance formation on the two alliance levels in Shark Bay remains unclear. Based on group-level relatedness patterns, previous work found that small second-order alliances consisted of more relatives than expected by chance, while a large alliance did not^45^. However, the alliances investigated in that study were the extremes in terms of size, but second-order alliance size in this population follows a continuum^41^. Furthermore, group-level analyses of relatedness are inherently problematic. Recent research at the individual level showed that associations of adolescent male dolphins correlated with relatedness and that the persistence of social bonds when males transitioned from adolescence into adulthood was determined by age similarity and association history, but not kinship^46^. During this transition, male dolphins increased their social network by forging bonds with similarly-aged but not necessarily related males. Thus, familiarity and age similarity appear more sought after traits than relatedness in alliance members.

Nevertheless, two crucial details about the ontogeny of male alliance formation remain unclear. First, it is unknown whether the previously reported absence of a kinship signal was merely due to the absence of relatives within the pool of potential allies as a result of slow life histories and single births^41^, or if males chose second-order alliance members independently of genetic relatedness. Second, marked first-order alliance partner preferences within second-order alliances have been described^42^, but the basis of such individual preferences has never been investigated. Since opportunities to mate with a female within consortships are shared among first-order alliance partners, males are expected to prefer relatives in order to maximise their evolutionary fitness. Alternatively, and similar to what is known in male chimpanzees^26–28^, male dolphins may prefer familiar but unrelated males with whom they share a social bond.

To investigate attributes influencing cooperative partner choice in male dolphins, we identified and characterised the entire pool of potential allies on an individual level. The aims were twofold: first, to compare certain traits (relatedness, social bond strength, age difference) between those males that were chosen as second-order alliance members with all those that were available but not selected during the adolescence period, when second-order alliances are not yet established; and second, to compare these traits between preferred and non-preferred first-order partners from within established second-order alliances during adulthood.

## Results

### Choice of second-order alliance members during adolescence

**Figure 1:**
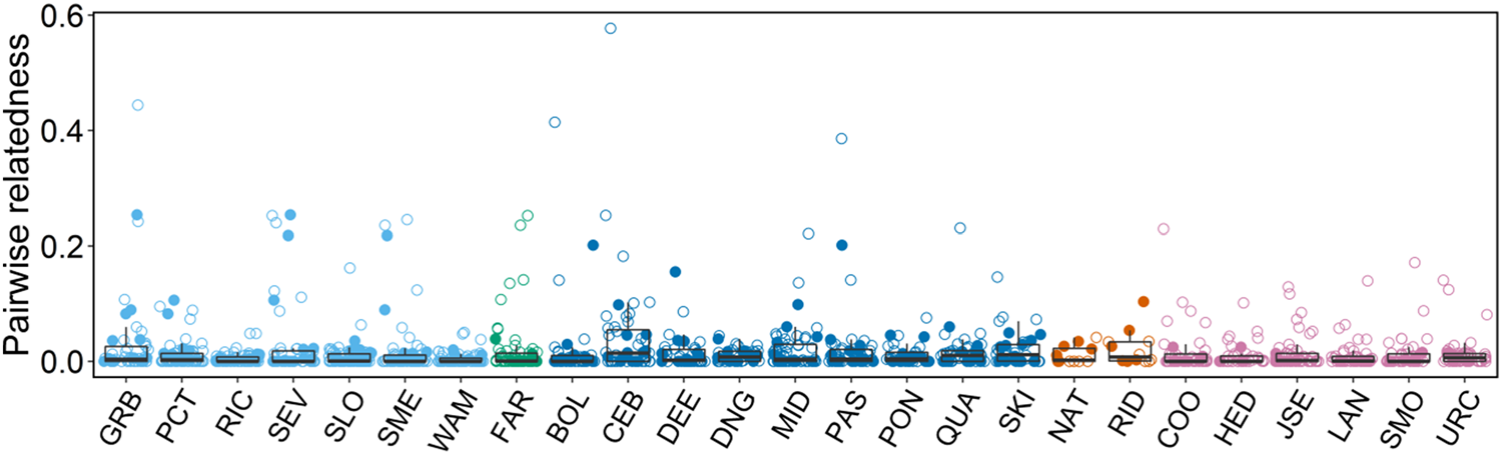
Distribution of dyadic relatedness values of each focal male used in the second-order GLMM with his chosen second-order alliance members and non-chosen males. Filled circles denote an individual’s chosen second-order alliance members, the non-filled circles non-chosen males. Colours correspond to second-order alliances. Boxplots represent the upper and lower quartiles ± 1.5 interquartile range as demarked by the whiskers.

We present data on 25 focal males belonging to five different second-order alliances (Supplementary Fig. S1 and Supplementary Table S1). The focal males had an average of 54.2 ± (14.3 s.d.) available but non-chosen males when they were adolescents and an average pool of 10.4 ± (3.4) second-order alliance members as adults (Supplementary Table S1). Non-chosen males consisted of all males that were old enough to form an alliance, were still in the population once the focal male reached adulthood and had overlapping home ranges with the focal males (“Methods”). Genetic data were available for all chosen alliance members and for 65.8% (± 14.7%) of non-chosen males. The non-genotyped males tended to have larger age differences from the focal males compared to those for which genetic data were available (mean age difference for non-genotyped males = − 10.85 ± 8.06 years, genotyped males = − 5.52 ± 7.57 years). Thus, father-son dyads might be missing in our dataset while most same-aged individuals can be identified. The mean relatedness of all focal males to their respective pool of chosen alliance members and non-chosen males was generally low (mean r = 0.0173 ± 0.0090, mean r chosen = 0.0172 ± 0.0120, mean r non-chosen = 0.0173 ± 0.0102, maximum r across all focal males = 0.2001 ± 0.1339, Fig. 1). For both the pools of chosen alliance members as well as non-chosen males, the focal males had, on average, less than one close relative (r ≥ 0.2) available (mean number of close relatives among chosen second-order members = 0.24, non-chosen males = 0.6).

**Figure 2:**
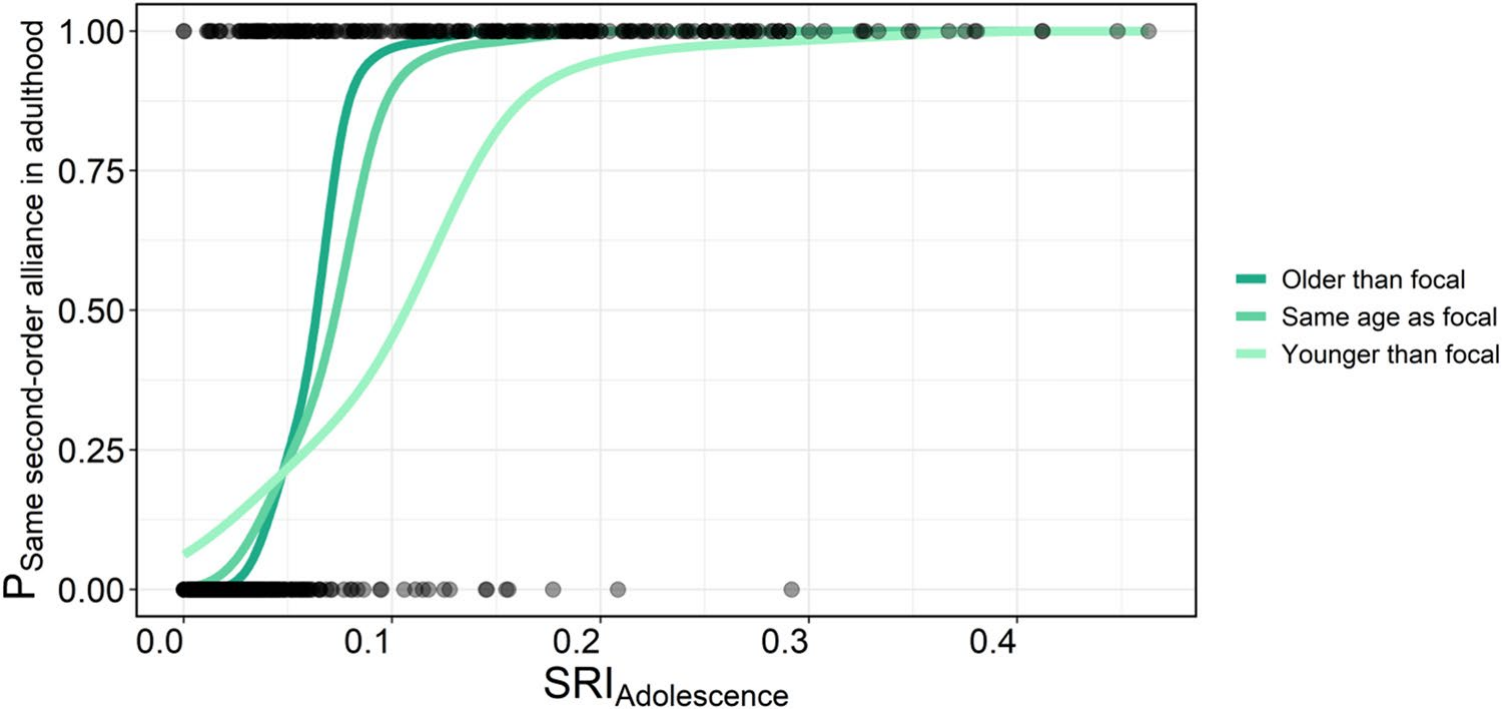
Interaction effect between social bond strength during adolescence (estimated from SRIs) and age difference on second-order alliance membership in adulthood. Focal males were more likely to be second-order alliance members in adulthood with males they associated with more often during adolescence, denoted by higher SRI values. However, older males and those of the same age were more likely to be chosen at lower SRI levels compared to younger males.

A binomial generalised linear mixed model (GLMM) was used to quantify the likelihood of second-order alliance formation between the 25 focal males and their respective pools of chosen alliance members and non-chosen males (second-order GLMM). The model indicated there was a significant interaction between the relative age difference and social bond strength during adolescence (estimated from Simple Ratio Indices (SRIs) which positively correlate with social bond strength^46^) between a focal and its potential allies (N = 1180, odds ratio = 2.53e−16, z = − 2.688, *p* = 0.007, Fig. 2, Table 1 and Supplementary Table S2). This suggests that the positive effect of social bond strength during adolescence on second-order alliance member choice was modulated by relative age, as similarly-aged and older potential members were more likely to be chosen by the focal at lower SRI values than younger potential members. Conversely, compared to the social bonds with older males, those with younger males had to be stronger for these younger males to be chosen as alliance members (Fig. 2). Generally, the majority of chosen second-order alliance members were of similar age to the focal males (Supplementary Fig. S2 and Supplementary Table S4). Further, our model showed that relatedness did not influence second-order alliance member choice (odds ratio = 3.08e−6, z = − 1.453, *p* = 0.146).

**Table 1:** Results from the GLMMs investigating second-order alliance member choice and first-order partner preferences.

	Exp(Β)		2.5%		97.5%		p-value	
	Second-order	First-order	Second-order	First-order	Second-order	First-order	Second-order	First-order
Intercept	1.34e−5	6.39e−3	4.60e−7	2.88e−3	3.88e−4	1.42e−2	**< 0.0001**	**< 0.0001**
Bond strength^a^	1.12e58	1.23e5	8.19e41	6.77e4	1.53e74	2.23e5	**< 0.0001**	**< 0.0001**
ΔAge	5.48	1.09	1.39	0.878	21.54	1.35	**0.015**	0.266
Relatedness	3.08e−6	2.82	1.13e−13	0.45	83.92	17.5	0.146	0.435
ΔAge^b^Bond strength	2.53e−16	n.a	1.08e−27	n.a	5.98e−5	n.a	**0.007**	n.a

Exponentiated fixed effects (Exp(B)) representing odds ratios, lower and upper confidence bounds (2.5% and 97.5%) and *p*-values of second-order GLMM and first-order GLMM investigating the effect of pairwise relatedness, age similarity, and SRI on choices of first-order partners and second-order members.

^a^SRI during the focal male’s adolescence for the model concerning second-order alliances, non-mating season SRI between males for first-order alliance partner choice.

Age^b^Bond strength denotes the interaction term between age difference and adolescence social bond strength on the level of second-order alliance member choice. Values in bold denote statistical significance (*p* < 0.05).

**Figure 3:**
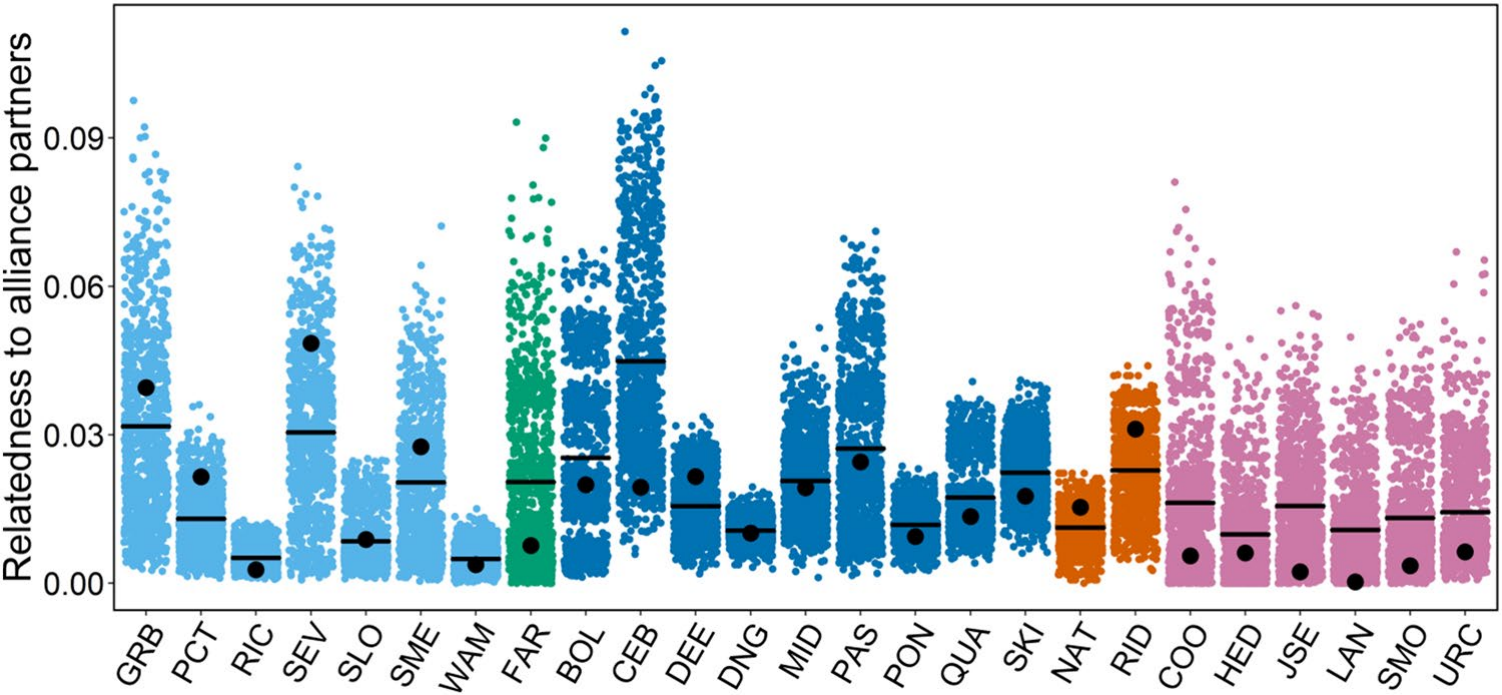
Scatter plot showing the distributions of the mean relatedness of focal males used in the second-order GLMM to randomly formed second-order alliances obtained from simulations. The mean of 1000 simulations per male is represented by the black line. The mean relatedness value of a focal male to its observed chosen second-order alliance members is displayed as a black circle. Different colours denote second-order alliance membership.

The finding that second-order alliance member choice is not kin-biased was supported by our simulations that aimed to investigate whether the mean relatedness of the focal males to their second-order alliance members was higher than expected by chance. The transformed mean relatedness of the individual focal males’ simulated alliances did not differ from the transformed mean relatedness of the individual focal males and their observed alliance members (two-tailed paired t-test, N = 25, t = − 1.26, df = 24, *p* = 0.2198, Fig. 3).

### Preferred and non-preferred first-order partners of adult males

**Figure 4:**
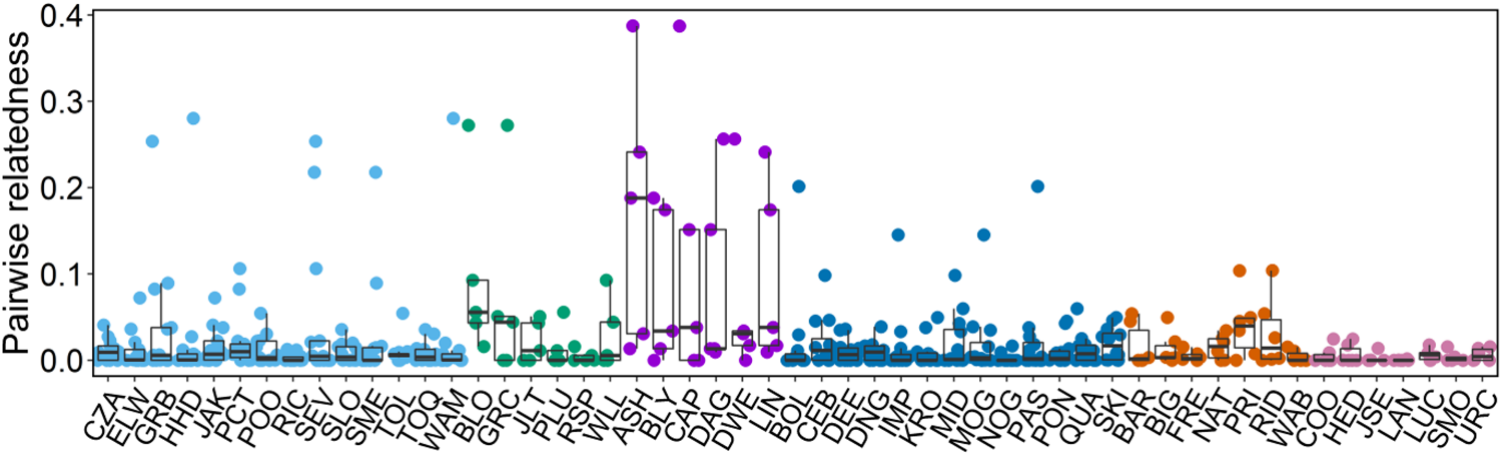
Distribution of relatedness values of 53 males to their second-order alliance members used in the first-order GLMM, which represent their available pool of males from whom to form first-order alliances. Individual data points indicating relatedness between the 53 males and their second-order alliance members are provided as filled circles, with colours representing second-order alliance membership.

To explore first-order partner preferences of adult males, we identified 53 well-known adult male dolphins (Supplementary Table S5). Each male was observed in 160 ± 93 (min = 46, max = 389) surveys on average, of which 54 ± 30 (min = 21, max = 140) surveys were conducted outside of the mating season. For these males, we tested whether non-mating season social bond strength, relatedness, and age similarity predicted first-order alliance partner preference during consortships within their respective second-order alliances. The 53 males were members of six different second-order alliances (Supplementary Fig. S1) for which genetic data were available for all members. Each of the males had on average 10.8 (± 3.2) second-order alliance members as potential first-order alliance partners (Supplementary Table S5). Average relatedness of the 53 males to their second-order alliance members was low (r = 0.023 ± 0.051, Fig. 4).

A GLMM on the first-order alliance level (first-order GLMM) revealed that partner preferences of adult males were significantly affected by social bond strength in the non-mating season, as shown by the positive correlation between non-mating season SRIs and the denominator of joint versus separate consortships (N = 482, odds ratio_SRI_ = 1.23e5, z_SRI_ = 38.622, *p*_SRI_ < 0.0001, Table 1 and Supplementary Table S3). Relatedness and age similarity did not influence the denominator of joint and separate consortships (odds ratio_kin_ = 2.82, z_kin_ = 1.112, *p*_kin_ = 0.266, odds ratio_age_ = 1.09, z_age_ = 0.781, *p*_age_ = 0.435) and, thus, did not affect partner choice on the first-order alliance level. All interactions were non-significant and therefore not included in the first-order GLMM model reported here.

## Discussion

We employed an individual-based approach to investigate partner choice on two levels of alliance formation spanning two age periods in male Indo-Pacific bottlenose dolphins. The main factor influencing choices at both the first- and second-order alliance levels was social bond strength. Social bond strength during adolescence foreshadowed second-order alliance memberships in adulthood and was of particular importance when future allies were younger than the focal males. In adult male dolphins, social bond strength during the non-mating season influenced first-order alliance formation, whereby males with stronger bonds outside of the mating season tended to favour consorting together during the mating season. As opposed to what is observed in many other species^47^, relatedness between males did not affect choices at either alliance level, while age similarity influenced the choice of second-order members only (Table 1).

Social bond strength appears pivotal in influencing the choice of allies across both alliance levels. This is expected if cooperation among males is not based on kin-selection but other mechanisms, such as by-product mutualism or reciprocity^48^. During bond formation, individuals have ample time to acquire information about the compatibility and reliability of potential allies. Compared to female dolphins, males invest more time in social activities from an early age^49^, suggesting that early social bond formation is crucial for males. Social bond strength is generally linked to cooperation across taxa^18,50,51^, and appears to predict the formation of male coalitions^19,52,53^. During adolescence, male-male encounters may enable the assessment of each other’s quality and compatibility as future allies, and promote competition over the best possible allies.

Our results suggest that males prefer those individuals with whom they were closely bonded during adolescence as second-order alliance members, perhaps because they had established a high degree of familiarity and compatibility^54,55^. Social bonds forged during adolescence may facilitate cooperative herding behaviours between second-order alliance members in adulthood, since males engage in consortship-like behaviours, play-herding for example, when adolescent^34^. Similarly, non-mating season social bond strength correlated with first-order partner preference in adulthood, suggesting a greater propensity to cooperate in risky tasks with closely bonded males, as previously documented in male chimpanzees^56^. During the mating season, adult male dolphins fight with males from other alliances over access to females, which entails risk of injury^41,57^.

The concept of homophily, i.e., the propensity to form social bonds with individuals of similar phenotypes, is useful for understanding the pattern of social bond formations in many animal societies. Homophily in age or rank^58–61^, relatedness^47^, tool use^62^, and personality^63^ can all influence social bond strength. We found that the majority of chosen second-order alliance members were of similar age to the focal male. Age-based patterns of association are observed across many taxa, from blacktip reef sharks (*Carcharinus melanopterus*)^64^ to chimpanzees^65^. Frequent associations among individuals close in age may stem from shared social interests^65^, such as the need for a play partner in adolescence or a coalition partner once adult, and similar energy budgets^66^. Considering the durability of second-order alliances, shared interests due to similar physical needs might facilitate the maintenance of cooperation and reduce the number of conflicts between alliance members. Indeed, common interests are hypothesised to limit cheating and exploitation among cooperating individuals^67^, thereby leading to stable patterns of cooperation.

Focal males showed a preference for older or same-aged males, reflected in the lower bond strength prerequisite to choosing them as second-order alliance members compared to younger males. Young males may have less consortship experience and likely lack the physical strength of older males. Males might choose younger males as alliance members only if older or same-aged males are not available, and only those with whom they are familiar. However, as in chimpanzees^68^, older males might be desired as second-order members but are likely scarce commodities, as the majority will already belong to a second-order alliance. It is unlikely that such males would leave their second-order alliance members to join a younger male, or that established second-order alliances will take up less experienced adolescent males. However, old males that have lost alliance members may be available. Indeed, such males have been observed joining younger males that were coalescing into a second-order alliance rather than remaining alone^41^. With adult males already belonging to second-order alliances and younger males even less experienced than the adolescent focal males, adolescents might not have options other than forming alliances with similar-aged males in need of alliance members. Therefore, our finding that males form second-order alliances with similarly-aged males might be explained by the population’s demography.

Relatedness did not influence first-order partner preference or second-order member choice on an individual level. This finding was supported by our simulations, which suggested that the average relatedness values of random alliances did not differentiate from those of the observed alliances. The finding of an earlier study, that small, stable first-order alliances appeared to be based on relatedness^45^, used a much more limited dataset than this study and was not supported here. The fact that relatedness does not influence partner preferences may seem surprising as male dolphins cooperate in order to gain fertilisations, an indivisible resource^29^. However, a species’ social system and the population’s demography may not allow for kin-based cooperation^69^. This has been observed in male chimpanzees^65^, male Galapagos hawks (*Buteo galapagoensis*)^70^, and cooperatively breeding choughs (*Corcorax melanorhamphos*)^71^, in which relatives were preferred cooperative partners but not always available. In dolphins, demographic constraints due to single births^43^, long interbirth intervals and a lack of reproductive skew at the population level^40^ result in the availability of few close male relatives. This is also supported by the low mean relatedness we found between the focal males and their available allies at both alliance levels. Interestingly, and in contrast to what is observed in chimpanzees, male dolphins did not prefer close relatives as allies (r ≥ 0.2), even when available. Furthermore, we know of at least three cases in which successive maternal brothers are in different second-order alliances (unpublished data). Males will maintain already developed social bonds even when a maternal half-brother becomes available, suggesting that joint skill development may override kinship.

In species that give birth to multiple offspring at once, or with high reproductive skew, multiple individuals share a set of half-siblings through either the maternal or paternal line which can be recognised through familiarity when phenotypic mechanisms to discriminate kin from non-kin are absent^72^. A larger number of shared half-siblings facilitates kin-biased alliance formation e.g., littermates in cheetahs (*Acionyx jubatus*)^21^. The long interbirth intervals^43^ and highly promiscuous mating system in Shark Bay^41^ means that individual dolphins are unlikely to have many close relatives available that can be recognised through familiarity, impeding the formation of kin-biased polyadic alliances. Even if relatives could be recognised and individuals could form dyadic alliances with kin to gain indirect fitness benefits, these may be offset by the direct benefits gained through the formation of larger, polyadic alliances with non-kin. Evidence that relatedness becomes negligible in the context of higher level, polyadic cooperation, potentially resulting from an insufficient number of relatives, can also be found in both chimpanzees and humans, where relatives are preferred partners in dyadic but not polyadic settings^73,74^.

In Shark Bay dolphins, the combination of a promiscuous mating system and low paternity skew likely result in male dolphins having an incongruent set of relatives (e.g., A’s half-brothers B and C are not necessarily half-brothers themselves). Due to the polyadic nature of alliances, this poses a ‘stable roommate problem’, where not every individual’s partner preferences can be met^75^, and where partner preferences are not fully independent. Male dolphins may thus ally with individuals they did not choose themselves, but which were chosen by other males belonging to the same alliance. Although we investigated partner choice on an individual level, we were unable to distinguish between males chosen by the focal male and those chosen by his allies. Despite this potential limitation, our results suggest that cooperative partner choice is a directed, non-stochastic process and, due to the impact on fitness, male dolphins in Shark Bay value those with whom they share the strongest bonds during adolescence as allies in adulthood, independently of relatedness. Our findings bear striking analogies to what is known on polyadic cooperation in chimpanzees and humans. Owing to this, our results imply that cooperation among non-kin is not unique to primates but a common feature of complex societies, marine and terrestrial alike.

## Methods

### Study population

Our study is based on long-term behavioural and genetic data collected on wild dolphins in eastern Shark Bay, Western Australia. Data collection in the form of boat-based surveys on this population started in 1984^41^. A ‘survey’ is a minimum 5-min observation of group size and composition, as well as predominant behaviour and GPS location^44^. Tissue samples for genetic analyses have been obtained regularly since 1997 using a remote biopsy system designed for small cetaceans^76^.

In this study, we were interested in how relatedness, age and social bond strength differ between individual males and their chosen allies versus their pool of potential but non-chosen males from two age periods: (i) adolescence, when males are 8–14 years old and second-order alliances are first formed from their pool of available males; and (ii) adulthood, when males are 15 years and older and successfully consort females in first-order alliances from within their established second-order alliances.

Permits for the scientific use of animals were obtained from the Department of Biodiversity, Conservation and Attractions, Western Australia. The University of Zurich and University of Western Australia granted animal ethics approvals. Behavioural and genetic data collection, as well as all methods in this study, were performed in accordance with the relevant guidelines and regulations. No direct handling/involvement of animals is involved in the study.

### Identification of chosen second-order alliance members and non-chosen males

The identification of second-order alliances and their constituent members was crucial to addressing the aims of our study. Male alliances are defined both by their association indices and their functional behaviour, cooperating in the herding and defence of females^77^. To confirm second-order alliance membership of adult males, we calculated association indices and carried out a hierarchical clustering analysis as described in Ref.^77^. Further information on the identification of second-order alliances and their members is provided in the Supplementary Information.

We calculated age difference, home range overlap and association rates for 25 ‘focal males’ for which we knew second-order alliance membership as adults. We based these analyses on their time as adolescents, i.e., before alliances were formed, enabling us to identify the individual pools of non-chosen males through that time period. This also allowed us to compare traits of non-chosen males to those of their chosen second-order alliance members. To quantify association rates, we calculated Simple Ratio Indices (SRIs) based on 5-min survey data in the R environment V3.6.2^78^ using *asnipe*^79^. Dyadic SRI values can range from 0 to 1. Individuals that are never seen in association are denoted by an SRI = 0, while a value of 1 indicates that two individuals were always observed together. We only included males with at least 20 survey records, independent of whether they were a focal male or not. Information on SRI calculations and age estimations is detailed in Ref.^42^.

We excluded males from the pool of non-chosen males if they were more than eight years younger than the focal or had disappeared before the focal male reached adulthood. We also excluded males that were never seen in association (SRI = 0) and had a home range overlap of less than 30% with the focal male (see Supplementary Information for details on this restriction).

### Choice of second-order alliance members during adolescence

We built a binomial generalised linear mixed model (GLMM) in R using *lme4*^80^ to test if the choice of second-order alliance members by the focal males, once adult, was influenced by relatedness, age difference or social bond strength (SRI) during adolescence (Supplementary Table S2, second-order GLMM). The units of analysis were dyadic measures between the focal males and their individual pools of chosen second-order alliance members, and the focal males and their non-chosen but available partners. Not all males included as actual or potential partners were themselves included as focal males. In contrast to our previous study^46^, we entered relative age differences instead of absolute age differences into our model. This allowed us to test if focal males consistently preferred older (age difference in years is negative) or younger (age difference is positive) males. To explore the effect of relatedness on the choice of second-order alliance members, we estimated pairwise relatedness between the focal males and their chosen second-order alliance members and the focal males and their non-chosen males from 9991 high-quality biallelic single nucleotide polymorphisms (SNPs). Pairwise relatedness estimates were calculated using the TrioML estimator^81^ in *Coancestry* V1.0.1.9^82^. A detailed laboratory protocol including bioinformatics filtering steps is provided in the Supplementary Information.

In the second-order GLMM, we included whether males were chosen as second-order alliance members as adults or not as a dichotomous dependent variable (yes/no). Social bond strength during adolescence, age difference, and pairwise relatedness between the focal males and their chosen members and the focal males and non-chosen males were entered as explanatory variables, including any interactions among them. To achieve model convergence and to facilitate the calculation and interpretation of interaction terms, we applied the ‘scale’ function in R on the age differences that ranged from − 30 to + 8 years. We subdued the very large positive skew in relatedness values, which spanned several orders of magnitude, by adding 1 followed by a log-transformation. To account for the dependency structure of our sample (dyadic data with repeated measures on the focal males and their potential and chosen members), we included the focal male’s individual ID code and the ID codes of chosen members and non-chosen males as random effects in the model. Summary statistics, including the *p-*values for statistical significance from Wald Z-tests, were obtained using the *car* package^83^.

In addition to the second-order GLMM, to assess in more detail whether males chose their second-order alliance members based on relatedness, as predicted by kin selection, we modelled random second-order alliances and compared mean relatedness values of focal males in these random alliances to their actual values based on their chosen second-order alliance members (R script provided in Supplementary Information). For each focal male, we simulated 1000 possible second-order alliances equal in size to its observed number of second-order alliance members. We did this by randomly drawing from the male’s pool of potential members (e.g., 1000 sets of six randomly drawn males for a male that had six observed second-order alliance members). Subsequently, we calculated the mean relatedness of the focal males to their randomly chosen second-order alliance members for each of the 1000 simulated alliances, as well as to their chosen second-order alliance members. Lastly, we log-transformed the simulated values after having added 1 and compared the mean of the averaged and transformed simulated relatedness values to the observed and transformed mean using a two-tailed paired t-test.

### Preferred and non-preferred first-order partners of adult males

We identified preferred and non-preferred first-order partners by calculating how often a male consorted with a specific second-order alliance member (joint consortships) and how many times they did not (separate consortships). We were interested in whether males had the strongest social bonds to their preferred first-order alliance partners outside the mating season. Male dolphins are observed together year-round but consortships peak during the mating season (Supplementary Fig. S3). To minimise the inclusion of sightings which may be connected to consortships, as well as to investigate the investment into social bonds prior to the occurrence of consortships, we calculated non-mating season SRIs among second-order alliance members from survey data collected between January and July (2001–2018). We thereby deliberately excluded the consortship peaks between August and December^84^ (Supplementary Fig. S3). We had to exclude consortships collected in 2009, 2012 and 2018 because non-mating season data was not collected during these years. As consortships can occur all year, we further excluded all surveys that were connected to any known consortship outside the mating season. Thus, we excluded consortship associations to measure social bond strength to the best of our ability.

We built a binomial GLMM in which we entered the binomial denominator consisting of the number of joint and separate consortships between second-order alliance members as a dependent variable (Supplementary Table S2, first-order GLMM). Explanatory variables were non-mating season SRIs, pairwise relatedness, and relative age difference in years. Random effects included the focal male’s ID code, the ID code of their second-order alliance members, as well as the second-order alliance code. Scaling and transformation of data and calculations of *p*-values were carried out as described for the second-order GLMM.

## Supplementary Information

Supplementary Information.

## Data Availability

The dataset(s) supporting the conclusions of this article are available on DRYAD.
