# Supplementary material for: Cooperative partner choice in multi-level male dolphin alliances
Source: Sci Rep. 2021 Mar 25;11:6901. doi: 10.1038/s41598-021-85583-x (PMC7994371; doi:10.1038/s41598-021-85583-x)
Supplement: Supplementary file 1 — Supplementary Information. [file 41598_2021_85583_MOESM1_ESM.docx]

Cooperative partner choice in multi-level male dolphin alliances

Livia Gerber*^a^, Samuel Wittwer^a^, Simon J. Allen^a,b,c^, Kathryn. G. Holmes^c^, Stephanie L. King^b,c^, William B. Sherwin^d^, Sonja Wild^e,f^, Erik P. Willems^a^, Richard C. Connor^g^, Michael Krützen^a^

1. Evolutionary Genetics Group, Department of Anthropology, University of Zurich, 8057 Zurich, Switzerland
2. School of Biological Sciences, University of Bristol, Bristol BS8 1TQ, United Kingdom
3. School of Biological Sciences and Oceans Institute, University of Western Australia, Crawley,

Western Australia 6009, Australia

1. Evolution and Ecology Research Centre, School of Biological, Earth and Environmental Sciences, UNSW Sydney, Sydney, NSW 2052, Australia
2. Centre for the Advanced Study of Collective Behaviour, University of Konstanz, 78464 Konstanz, Germany
3. Cognitive and Cultural Ecology Research Group, Max Planck Institute of Animal Behavior, 78315 Radolfzell, Germany
4. Biology Department, UMASS Dartmouth, North Dartmouth, MA 02747, USA

Corresponding author: Livia Gerber, Evolutionary Genetics Group, Department of Anthropology, University of Zurich, 8051 Zurich, Switzerland. Phone: +41 635 54 32. Email: [livia.gerber@aim.uzh.ch](mailto:livia.gerber@aim.uzh.ch)

## Supplementary Information

## Second-order alliance identification and members

Second-order alliance membership was confirmed by calculating Simple Ratio Indices (SRI) based on five-minute survey data and a hierarchical clustering analysis (as described in 42) as well as by documenting consortships between the males (consortships were scored based on established criteria, as outlined below). For the purpose of this study, we identified and confirmed alliance membership for a total of 58 males belonging to six second-order alliances with variable alliance sizes of 6-14 individuals (see Figure S1). We had to exclude five alliance members (BAK, EXF, FAR, JAA, TER) from the analyses investigating first-order alliance partner choice because they were seen fewer than 20 times in the non-mating seasons between 2001 and 2018 and, thus, their non-mating season associations were considered unreliable. Furthermore, we could not estimate the year of birth for JAA.


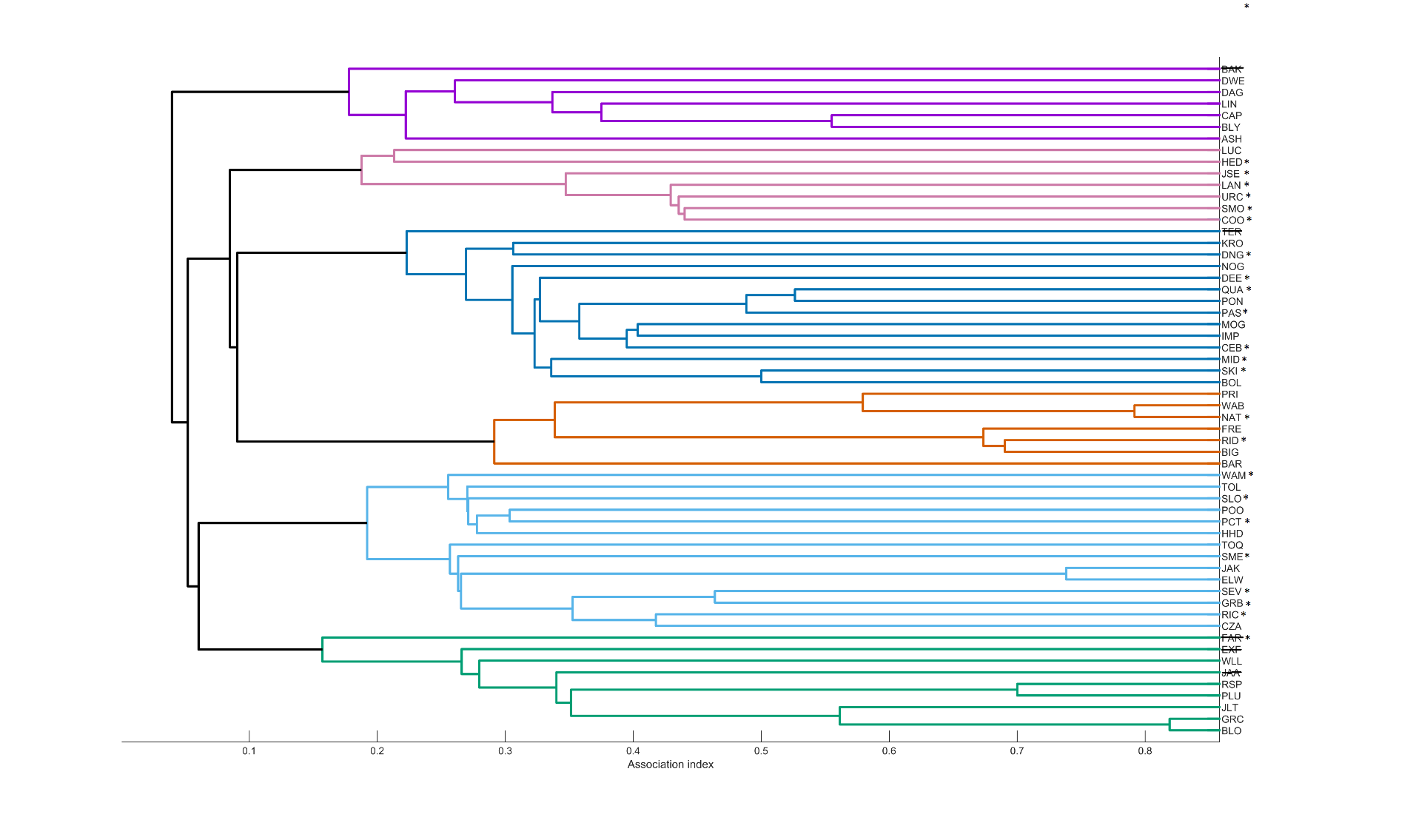


Figure S1: Dendrogram of the second-order alliances identified functionally via consortship data and confirmed via hierarchical clustering analysis based on survey data. Males included in the second-order GLMM are marked with an asterisk (*). In the first-order GLMM, all males were included apart from the ones striked-through.

## Shark Bay Ethogram – Consortship Criteria

Consortships are coercively maintained associations between 2-3 males and one female. Consortships are scored as YES (Y), NO (N), or PROBABLE (P) for both the male subgroup and suspected female consort. Scoring the consortship as a YES for both males and female consort requires satisfaction of the following criteria:

1. The subgroup of 2-3 males + 1 female is observed with >10m separation between any other individual or group, AND
2. ONE or more of the following 6 events are observed:
   1. **1hr:** Consortship subgroup is observed for at least 1 hour. At least one male from the consortship subgroup is within 10m of the suspected female throughout the 1hr observation period.
   2. **Capture:** The female is captured by an alliance of males.
   3. **Bolt:** Female attempting to escape by rapidly swimming ('bolting') from an alliance of males.
   4. **Pops:** At least one of the males producing a vocal threat called 'pops' that induces the female to remain close.
   5. **Directed Aggression:** At least one of the males directing physical threats or aggression toward the female. Aggressive behaviour described in the Shark Bay Dolphin Research Ethogram.
   6. **Theft:** Teams of two alliances attempting to take a female from another alliance. In this case, the consortship is scored as a ‘Y’ for losing males, winning males, and female.

If A observed but B is not observed, the consortship should be scored as a ‘P’ if:

- > 10m separation, one female, >30 min but < 1hr observation (‘P’ for males & female)

| - Focal ID | Number of non-chosen males / thereof sampled | Number of chosen second-order alliance members |
| --- | --- | --- |
| BOL | 52 / 25 | 13 |
| CEB | 54 / 34 | 13 |
| COO | 50 / 39 | 6 |
| DEE | 53 / 25 | 13 |
| DNG | 49 / 23 | 13 |
| FAR | 67 / 57 | 7 |
| GRB | 55 / 40 | 13 |
| HED | 53 / 36 | 4 |
| JSE | 58 / 43 | 6 |
| LAN | 51 / 40 | 6 |
| MID | 75 / 48 | 13 |
| NAT | 14 / 6 | 6 |
| PAS | 54 / 30 | 13 |
| PCT | 61 / 51 | 12 |
| PON | 50 / 22 | 13 |
| QUA | 60 / 35 | 13 |
| RIC | 69 / 54 | 13 |
| RID | 14 / 6 | 6 |
| SEV | 54 / 39 | 13 |
| SKI | 52 / 25 | 13 |
| SLO | 79 / 63 | 13 |
| SME | 54 / 44 | 13 |
| SMO | 47 / 37 | 6 |
| URC | 64 / 44 | 6 |
| WAM | 64 / 55 | 12 |

Table S1: Summary of the 25 focal males to investigate second-order alliance member choice. The table provides each focal male’s ID code, their number of potential, non-chosen males and how many thereof were genotyped. The last column indicates each focal male’s number of actual, chosen second-order alliance members.

## Model specifications

| **Model** |  |
| --- | --- |
| 1 | second-order members y/n ~ r + Δage*SRI + (1\|ID_F_) + (1\|ID_C_) |
| S1 | second-order members y/n ~ Δage + Δage^2^ + (1\|ID_F_) + (1\|ID_C_) |
| 2 | separate : joint consortships ~ SRI^†^+ Δage + r + (1\|ID) + (1\|ID_P_) + (1\|Alliance) |

Table S2: Model specifications for Models 1 (second-order GLMM) and 2 (first-order GLMM) investigating partner choice on the second-order and first-order alliance level. Of all possible interactions, only the one between age difference and social bond strength on the level of second-order member choice was significant and thus, included. Both binomial GLMMs included relative age difference (Δage), social bond strength (SRI), and relatedness (r). We log-transformed the relatedness values after adding 1 and applied the ‘scale’ function to the age difference before entering them into the Models.

The second-order GLMM investigated whether two males became members of the same second-order alliance or not (second-order members y/n). Social bond strength in the second-order GLMM was the SRI between the focal and a male from its cast during the focal male’s adolescence. The * denotes the interaction effect between age difference and social bond strength. The random effects of the second-order GLMM consisted of the three letter ID codes of the focal male (ID_F_) and the males from their cast, consisting of chosen alliance members and non-chosen males (ID_C_).

Model S1 is a post-hoc GLMM analysis based on the results of the second-order GLMM in which we investigated if males were more likely to form second-order alliances with similarly-aged males. Model S1 contains relative age difference as linear variable as well as its quadratic term. Also here, the scale function was applied on Δage.

In the first-order GLMM, the binomial denominator consisted of how often two second-order alliance members were observed in joint or separate consortships (separate : joint consortships), allowing us to explore first-order alliance partner choice. In this model, social bond strength (SRI^†^) was the non-mating season SRI between second-order alliance members and included second-order alliance membership (Alliance) as a random factor.

## Detailed results of first- and second-order GLMMs

|  | Β | | S.E. | | z-value | | p-value | |
| --- | --- | --- | --- | --- | --- | --- | --- | --- |
|  | second-order | first-order | second-order | first-order | second-order | first-order | second-order | first-order |
| Bond strength^†^ | 133.663 | 11.718 | 18.957 | 0.303 | 7.051 | 38.622 | **<0.0001** | **<0.0001** |
| Relatedness | 1.701 | 0.086 | 0.698 | 0.110 | 2.437 | 0.781 | 0.146 | 0.266 |
| ΔAge | -12.690 | 1.037 | 8.735 | 0.932 | -1.453 | 1.112 | **0.015** | 0.435 |
| ΔAge*Bond strength | -35.911 | n.a. | 13.361 | n.a. | -2.688 | n.a. | **0.007** | n.a. |

Table S3: Results of the second-order GLMM and first-order GLMM (Table S1), investigating the effect of pairwise relatedness, age similarity, and SRI on ally choice on the first- and second-order level of male alliances. ^†^SRI during the focal male’s adolescence for the model concerning second-order alliances, non-mating season SRI between for first-order alliance partner choice. Age*Bond strength denotes the interaction term between age difference and social bond strength on the level of second-order alliance member choice.

## Results of Model S1

To test if males chose similarly-aged males as second-order alliance members which could not be inferred from the second-order GLMM due to non-convergence, we ran a post-hoc GLMM (Model S1) in which we entered age differences as a quadratic as well as a linear term. The significant effect of the quadratic term suggests that focal males primarily formed second-order alliances with their peers (N = 1,180, Table S3, Figure S2).

|  | Β | S.E. | z-value | p-value | C^Β | 2.5% | 97.5% |
| --- | --- | --- | --- | --- | --- | --- | --- |
| Intercept | -3.180 | 0.443 | -7.174 | **<0.0001** | 0.042 | 0.02 | 0.10 |
| ΔAge^2^ | 0.975 | 0.385 | 2.535 | **0.0111** | 2.65 | 1.24 | 5.63 |
| ΔAge | -1.751 | 0.487 | -3.596 | **0.0003** | 0.17 | 0.066 | 0.45 |

Table S4: Results of Model S1 investigating if males chose similarly-aged or older males as alliance members. The significant effect of the quadratic age difference indicates that focal males were more likely to form second-order alliances with similarly-aged males.


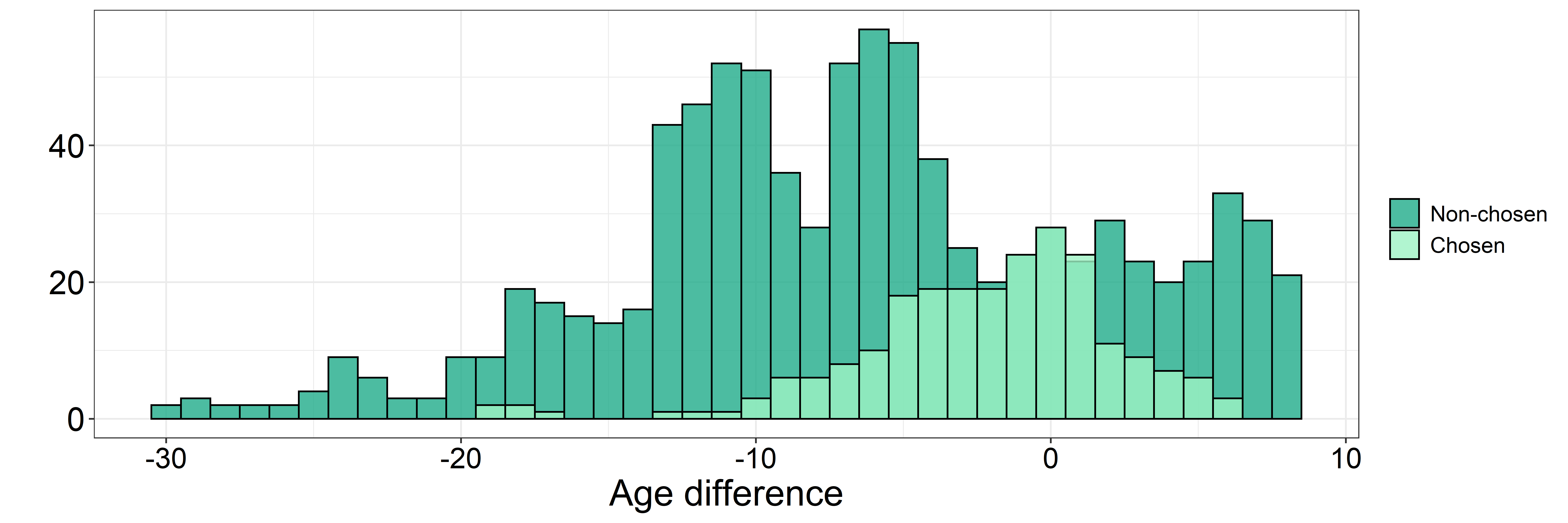
Figure S2: A large proportion of chosen alliance members were of similar age to the focal, while males with larger age differences were chosen less often.

## Home range overlap of second-order alliance members

The smallest observed home range overlap between adolescent males who became actual second-order alliance members was 0.36. Slightly more conservative, we used 0.30 as a cut-off to exclude males as potential members based on their home range overlap. However, males with home range overlaps smaller than 0.30 but who were observed in association with the focal male at least once during the focal male’s adolescence were still treated as potential alliance members for second-order alliance partner choice.

## Seasonality of consortships


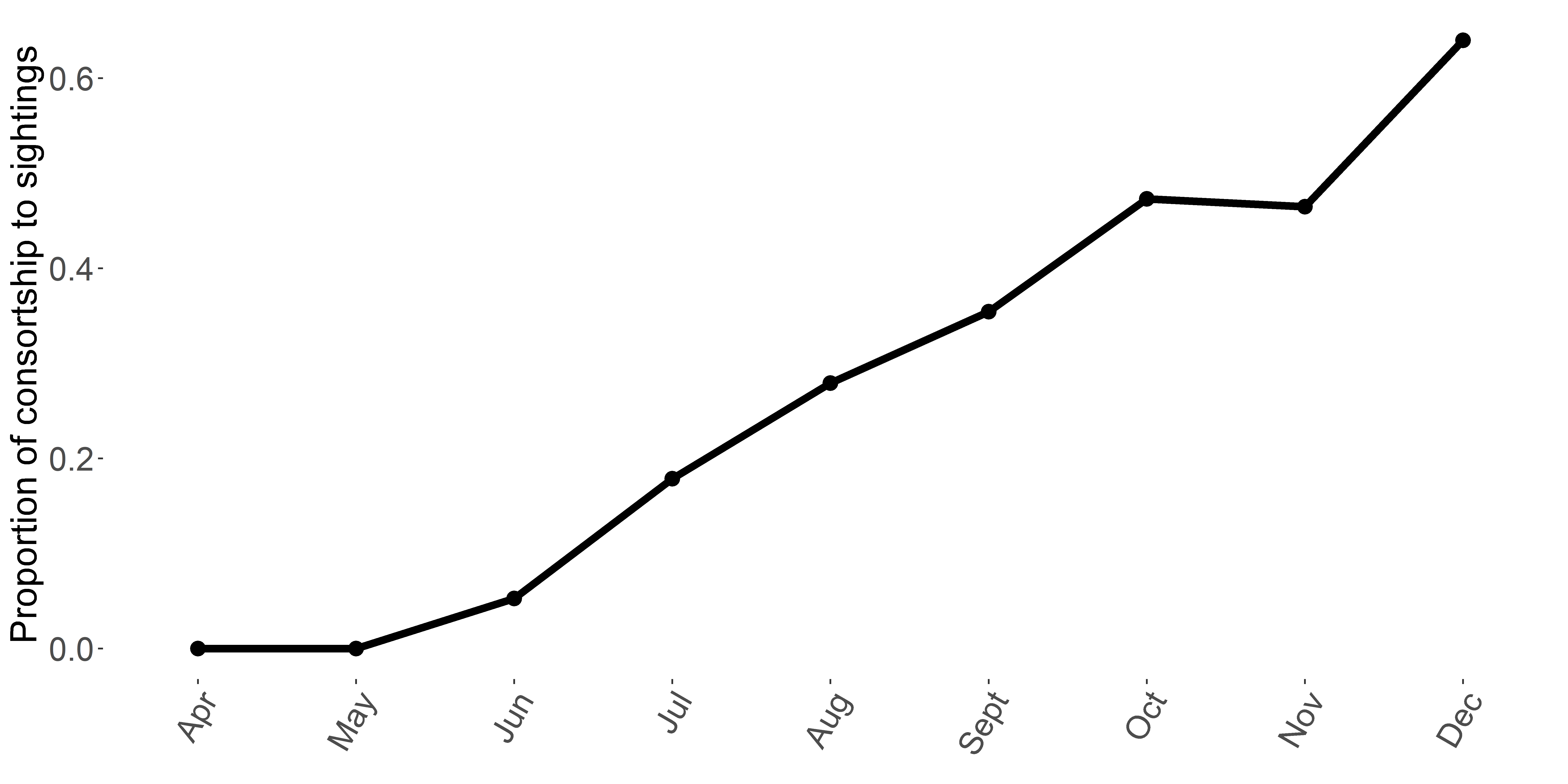


Figure S3: Line plot visualizing the increase of consortships at the start of the mating season in August. The Y-axis contains the proportion of consortship to non-consortship sightings (during surveys) of male dolphins.

| second-order alliance | ID focal | Number of potential first-order alliance partners |
| --- | --- | --- |
| BL | CZA | 13 |
| BL | ELW | 13 |
| BL | GRB | 13 |
| BL | HHD | 13 |
| BL | JAK | 13 |
| BL | PCT | 13 |
| BL | POO | 13 |
| BL | RIC | 13 |
| BL | SEV | 13 |
| BL | SLO | 13 |
| BL | SME | 13 |
| BL | TOL | 13 |
| BL | TOQ | 13 |
| BL | WAM | 13 |
| XF | BLO | 8 |
| XF | GRC | 8 |
| XF | JLT | 8 |
| XF | PLU | 8 |
| XF | RSP | 8 |
| XF | WLL | 8 |
| HC | ASH | 6 |
| HC | BLY | 6 |
| HC | CAP | 6 |
| HC | DAG | 6 |
| HC | DWE | 6 |
| HC | LIN | 6 |
| KS | BOL | 14 |
| KS | CEB | 14 |
| KS | DEE | 14 |
| KS | DNG | 14 |
| KS | IMP | 14 |
| KS | KRO | 14 |
| KS | MID | 14 |
| KS | MOG | 14 |
| KS | NOG | 14 |
| KS | PAS | 14 |
| KS | PON | 14 |
| KS | QUA | 14 |
| KS | SKI | 14 |
| PD | BAR | 5 |
| PD | BIG | 5 |
| PD | FRE | 5 |
| PD | NAT | 5 |
| PD | PRI | 5 |
| PD | RID | 5 |
| PD | LUCWAB | 5 |
| RR | COO | 6 |
| RR | HED | 6 |
| RR | JSE | 6 |
| RR | LAN | 6 |
| RR | LUC | 6 |
| RR | SMO | 6 |
| RR | URC | 6 |

Table S5: Overview of the 53 adult focal males used to investigate first-order alliance partner choice, including each focal male’s second-order alliance membership and size thereof, reflecting its pool of available second-order alliance members to choose as first-order alliance partners. All members of all second-order alliances have been genotyped successfully.

## Modelling second-order alliances to test influence of relatedness on partner choice

### MODEL OF RANDOM SECOND ORDER ALLIANCE CHOICE BASED ON SHARED RELATEDNESS ###

# LOAD FILE CONTAINING IDs (FOCAL IN COLUMN 1, POTENTIAL PARTNER ID IN COLUMN 2), R-VALUES BETWEEN IDs IN COLUMN 10, ACTUAL ALLIANCE SIZE OF FOCAL IN COLUMN 29, AVERAGE R-VALUE TO ACTUAL ALLIANCE PARTNERS IN COLUMN 30

FocalMalesSRI_LE_20190214_LMM_no0TrioML <- read.csv(YourPath:/FocalMalesSRI_LE_20190214_LMM_no0TrioML.csv", header=TRUE)

#USE AGGREGATE FUNCTION TO OBTAIN LIST CONTAINING ALL INDIVIDUALS AND THEIR SECOND-ORDER ALLIANCE SIZE

IDs <- aggregate(FocalMalesSRI_LE_20190214_LMM_no0TrioML[,30],list(FocalMalesSRI_LE_20190214_LMM_no0TrioML$ID1),mean)

#CREATE AGENTS (I.E. FOCAL MALES)

setup <- function(){

return(data.frame(id=c(IDs$Group.1), averageR=c(IDs$x)))

}

indDF <- setup()

#SPLIT UP DATASET AND CREATE A LIST CONTAINING SEPARATE DATAFRAMES OF ALL AGENTS AND THEIR POTENTIAL PARTNERS

potentialpartners <- split(FocalMalesSRI_LE_20190214_LMM_no0TrioML, FocalMalesSRI_LE_20190214_LMM_no0TrioML$ID1)

potentialpartners <- as.list(potentialpartners)

#EXTRACT R VALUES TO POTENTIAL PARTNERS (COLUMN3) AND NUMBER OF ACTUAL ALLIANCE PARTNERS (COLUMN 29) FOR EACH AGENT

ObtainrvaluesAA <- function(x){

x[,c(10,29)]

}

allRvaluesAndAA <- lapply(potentialpartners, ObtainrvaluesAA)

chooseAlly <- function(ind){

chosenPartner <- sample(x = ind$TrioML, size = mean(ind$AA), replace = FALSE) #randomly choose alliance partner

ind$partner <- mean(chosenPartner)

return(ind)

}

#EXTRACT AGENT’S ID AND AVERAGE R-VALUE OF RANDOM ALLIANCES FORMED OF EACH INDIVIDUALS ALLIANCE PARTNERS, FEED VALUES INTO SEPARATE LIST

Form1000Alliances <- replicate(1000,lapply(allRvaluesAndAA,chooseAlly))

x <- as.data.frame(do.call("rbind",lapply(Form1000Alliances,"[",1,3)))

tmp <- data.frame(

X = x$V1,

ind=rep(1:34,nrow(x)/34000) #34 = NUMBER OF AGENTS

)

Average1000Alliances <- unstack(tmp, X~ind)

y <- apply(Average1000Alliances,2,mean)

indDF["averageRandomChoice1000"] <- y

#CARRY OUT T-TEST TO SEE IF MEAN OF ALL AVERAGE R-VALUES OF 1000 RANDOM ALLIANCES DIFFERS FROM OBSERVED AVERAGE R-VALUE TO ALLIANCE PARTNERS

t.test(indDF$averageR,meansSimulated, paired = TRUE, alternative = "two.sided")

## Quality filtering to identify high-quality SNPs

To identify high-quality biallelic SNPs for accurate relatedness estimation, we aligned the quality filtered reads against a *T. truncatus* reference assembly (GenBank: GCA_001922835.1 (77) using bowtie2 version 2.2.6 (78) with the ‘very-sensitive’ preset. Variant calling was performed using GATK version 4.1.1.0 by first generating per-individual g.vcf files with HaplotypeCaller and subsequent joint variant calling using CombineGVCFs. We hard-filtered the resulting 54'854 raw SNP variants with vcftools (79) and obtained our final 9,991 biallellic SNP loci based on a phred quality score over 30, sequencing depth per locus of at least five, per locus coverage in over 50% of individuals, minor allele count of 3, and minimal distance between SNPs of 100kb.

## ddRAD laboratory Protocol

Genomic DNA was extracted from small tissue biopsy samples using the Gentra Puregene Tissue Kit (Qiagen). Subsequently, we quantified our DNA extracts using a Qubit 1.0 fluorometer with the Qubit dsDNA BR Assay Kit (ThermoFisher Scientific). We set up a restriction digest consisting of 250 ng of genomic DNA per individual and 20 units of MseI (New England Biolabs, ‘NEB’) as well as high-fidelity EcoRI (New England biolabs), followed by eluting the digested DNA twice in 18μl buffer using the MinElute PCR cleanup (Qiagen). To normalise the samples and to ensure even representation during sequencing, we used the Qubit with the dsDNA HS Assay Kit (ThermoFisher Scientific).

To each sample, we ligated differing EcoRI P1 barcode adapters in order to assign obtained reads to individuals after sequencing. MseI restriction ends were ligated to MseI P2 adapters containing degenerate bases, which allowed us to detect PCR duplicates after sequencing (80). All sequences of adapters, barcodes and primers used are provided in the SI. The ligation reaction per sample was set up in a total volume of 45μl and consisted of 400 Units of T4 Ligase (NEB), 4.5μl T4 Ligase Buffer (NEB), 1.5μl MseI adapter (10μM), 1.5μl EcoRI adapter (1μM), and 150ng DNA. The ligation took place in a Veriti Thermal Cycler (Applied Biosystems) set to 23°C for one hour, followed by 65°C for 10 minutes and was cooled to 4°C at a rate of 2°C per 90 seconds.

After the ligation, the now individually-barcoded samples were pooled and size-selected. We used three AMPure bead size selections. The first two to obtain fragments in the approximate range between 200 and 500 base pairs. For this, we added 0.65 volumes of AMPure bead mix to the pooled samples to remove larger fragments and added 0.16 volumes (of the original volume) to remove small fragments. In the third bead selection we added 1.2 volumes of AMPure beads, which ensured complete removal of the adapter dimers and eluted the DNA fragments in 28μl of ddH_2_O.

The size-selected DNA was then amplified via PCR. We set up ten 30μl reactions consisting of 1.5μl primer 1 and 2 each (10μM stock concentration), 0.9μl DMSO, 15μl Phusion Mastermix 2X (NEB), 8.7μl ddH2O, and 2.4μl DNA. The reactions were transferred to a preheated (98°C) thermocycler (Labcycler, SensoQuest). Followed by an initial denaturation of 98°C for 30 seconds, the DNA was amplified in ten PCR cycles (denaturation at 98°C for 10 seconds, annealing at 65°C for 30 seconds, extension at 72°C for 30 seconds). Post PCR, the reactions were pooled and cleaned up in a single MinElute PCR cleanup column including several loading steps and eluted twice in 18μl EB buffer, yielding 36μl of amplified product.

To ensure sequencing of homologous genomic regions across the different libraries, we carried out a last size selection by gel electrophoresis on Spreadex® EL600 gels (AL Diagnostics) as described in (81) with the following modifications: we loaded a 25μl amplified library in two separate lanes to avoid DNA overloading and ran the gel electrophoresis for 228 minutes at 120 Volt. We avoided UV exposure by visualizing DNA fragments on a blue light transilluminator (Dark Reader DR46B, Clare Chemical Research) and excised fragments between 307 and 404 bp. For DNA recovery using electro elution, the packages containing the excised gel fragments were placed in the electrophoresis chamber overnight at a buffer temperature of 20°C at 90V.

We produced a total of eight libraries, consisting of 40 samples each. The libraries were sequenced in the rapid run mode on an Illumina HiSeq2500, using one lane per library, combining two libraries per run. To distinguish between samples of the same barcode but different libraries, one library was ligated to MseI adapters with an Index 6 sequence, the other to Index 12 or Index 4 (see detailed laboratory protocol below for sequences).

### Step-by-step ddRAD wet lab protocol

Adapters: **Adapter P1 = *Eco*RI x 48**

**P1.1 (*Eco*RI top):**

5’ ACACTCTTTCCCTACACGACGCTCTTCCGATCTTCGAT 3’

**P1.2 (*Eco*RI bottom):**

5’ AATTATCGAAGATCGGAAGAGCGTCGTGTAGGGAAAGAGTGT 3’

| 1 | GCATG_EcoRI | 25 | CTGCG_EcoRI |
| --- | --- | --- | --- |
| 2 | AACCA_EcoRI | 26 | CTGTC_EcoRI |
| 3 | CGATC_EcoRI | 27 | CTTGG_EcoRI |
| 4 | TCGAT_EcoRI | 28 | GACAC_EcoRI |
| 5 | TGCAT_EcoRI | 29 | GAGAT_EcoRI |
| 6 | CAACC_EcoRI | 30 | GAGTC_EcoRI |
| 7 | GGTTG_EcoRI | 31 | GCCGT_EcoRI |
| 8 | AAGGA_EcoRI | 32 | GCTGA_EcoRI |
| 9 | AGCTA_EcoRI | 33 | GGATA_EcoRI |
| 10 | ACACA_EcoRI | 34 | GGCCA_EcoRI |
| 11 | AATTA_EcoRI | 35 | GGCTC_EcoRI |
| 12 | ACGGT_EcoRI | 36 | GTAGT_EcoRI |
| 13 | ACTGG_EcoRI | 37 | GTCCG_EcoRI |
| 14 | ACTTC_EcoRI | 38 | GTCGA_EcoRI |
| 15 | ATACG_EcoRI | 39 | TACCG_EcoRI |
| 16 | ATGAG_EcoRI | 40 | TACGT_EcoRI |
| 17 | ATTAC_EcoRI | 41 | TAGTA_EcoRI |
| 18 | CATAT_EcoRI | 42 | TATAC_EcoRI |
| 19 | CGAAT_EcoRI | 43 | TCACG_EcoRI |
| 20 | CGGCT_EcoRI | 44 | TCAGT_EcoRI |
| 21 | CGGTA_EcoRI | 45 | TCCGG_EcoRI |
| 22 | CGTAC_EcoRI | 46 | TCTGC_EcoRI |
| 23 | CGTCG_EcoRI | 47 | TGGAA_EcoRI |
| 24 | CTGAT_EcoRI | 48 | TTAACC_EcoRI |

Table containing barcode sequences to distinguish pooled samples bioinformatically after sequencing**.**

**Adapter P2 = *Mse*I**

**P2.1 (*Mse*I top):**

5’ /5Phos/TAGATCGGAAGAGCACACGTCTGAACTCCAGTCAC 3’

**P2.2 (*Mse*I bottom):**

Idx_6 GCCAAT

5’ CAAGCAGAAGACGGCATACGAGATNNNN**ATTGGC**GTGACTGGAGTTCAGACGTGTGCTCTTCCGATC 3’

Idx_12 CTTGTA

5’ CAAGCAGAAGACGGCATACGAGATNNNN**TACAAG**GTGACTGGAGTTCAGACGTGTGCTCTTCCGATC 3’

Idx_4 TGACCA

5’ CAAGCAGAAGACGGCATACGAGATNNNN**TGGTCA**GTGACTGGAGTTCAGACGTGTGCTCTTCCGATC 3’

**Illumina PCR primers**

**ILLPCR1**

5’ AATGATACGGCGACCACCGAGATCTACACTCTTTCCCTACACGACG 3’

**ILLPCR2**

5’ CAAGCAGAAGACGGCATACGA 3’

**See last page for an overview of the setup presented here**

#### Preparation of Specialised Reagents

*Adapter P1:* Barcoded *Eco*RI primer combinations.

- Combine 1μl P1.1 + 1μl P1.2 (100μM stock) with 98μl ddH_2_O to make 100ul of 1μM annealed, double stranded adapter stock.
- Heat to 95°C for 5 min and slowly cool to room temperature (no faster than 2°C/min).
- Keep the adapters organised in plate format for easy use in future directions
- Adapters can be kept at 4°C when in use but not longer than 2 weeks. For long term storage keep at -80°C.

*Adapter P2: Mse*I-bar adapter

- Combine 10μl of P2.1 + P2.2 (100μM stock) with 80μl ddH_2_O to make 100μl of 10μM stock.
- Heat to 95°C for 5min and slowly cool to room temperature to anneal oligos into double-stranded adapters (not faster than 2°C/min).
- Storage same as above.

*PCR primers*

1. Mix 50μl of each primer (100μM stock) with 900μl ddH_2_O to make a working solution containing 5μM of each primer.

- Storage same as above.

#### Sample Preparation

Measure DNA concentration

- Start with 15μg of DNA per library. For 50 samples this equates 300ng of DNA/sample, made up to 84μl with ddH_2_O.
- The cleanup/ligation needs to be carried out in a clean PCR plate (easier for AMPure cleanup)

#### Restriction Digest

Prepare Master Mix I

- 16μl per sample
- Use low binding Eppendorf tubes due to high viscosity of the enzymes.

| **Reagent** | **1x** | **1x (ul)** | **55x (ul)** |
| --- | --- | --- | --- |
| Cutsmart buffer (10x) | 1x | 1.6 | 88 |
| *Mse*I (10,000U/ml) | 20U | 2.0 | 110 |
| *Eco*RI (20,000U/ml) | 20U | 1.0 | 55 |
| ddH_2_O |  | 11.4 | 627 |
| ***Total*** |  | ***16*** | ***880*** |

- Mix by vortexing and centrifuge briefly.
- Add 16μl of MM1 to each well
- This gives a total of 100μl.
- Seal plate. Vortex and spin down.
- Digest at 37°C for 7hrs and cool to RT
- Do not heat kill as this skews base composition
- 20U of enzyme digests 1mg DNA in 30min, but DNA was found to not always be digested completely
- There is uneven digestion between samples
- Before moving to the next step, cool restriction digest to RT. Alternatively, product can be stored at 4°C overnight.

#### MinElute PCR Cleanup

The columns clean up the Restriction digest but don’t get rid of the adapter dimer. This needs to be cleaned with AMPure cleanup just before the size selection.

1. Follow the manufacturer’s instructions.
2. Elute twice in 18μl buffer for a total elute of 36μl (incubate for 5 minutes each time).
3. 1μl can be kept to run on Tapestation/gel

#### Normalise Samples

- Determine the DNA concentration of each cleaned digest using Qubit dsDNA BR (1μl).
- Normalise the samples (100-300ng/sample is optimal)

This step is essential for ensuring even representation of samples in the sequencing.

#### Adapter Ligation

Defrost *Eco*RI adapters (in plate form) and *Mse*I-bar adapters (in one eppie in the -80°C freezer) in the fridge or on ice.

- Make Master Mix II (MMII)

| **Reagent** | **1x** | **1x (μl)** | **55x (μl) Idx_XX** |
| --- | --- | --- | --- |
| T4 Ligase Buffer 10x | 1x | 4.5 | 247.5 |
| *Mse*I Adapter (10μM) |  | 1.5 | 82.5 |
| T4 Ligase (400 U/ml) |  | 1 | 55 |
| Water + DNA |  | 36.5 |  |
| *Total* |  | *45.0* | *7*μ*l/sample* |

- Add 7ul of MMII to each well containing 36.5μl DNA + water. Add 1.5μl of the *Eco*RI (1μM working solution) adapters to each corresponding sample well for a total volume of 45μl.
- Ligate in PCR cycler: 1hr @ 23°C. 10min @ 65°C. Cool to 4°C at a rate of 2°C/90sec.
- Keep 1μl to run on Tapestation

#### Pooling and Size Selection

Be aware that different fragments are obtained when carrying out the size selection in Lo-bind tubes

Before you continue:

- Leave AMPure beads on the bench for 30min to equilibrate to RT
- Make fresh 80% EtOH
- Pool half of all the individually barcoded DNA samples in one tube and mix well, do the same with the other half and carry out the bead selection on both tubes at the same time.

**First bead selection to remove large fragments**

- Add 0.65 volumes AMPure bead mix to the pooled samples (e.g., 65μl for a volume of 100μl).
- Mix by pipetting up and down at least 10 times.
- Incubate on the bench for 10-15min at RT.
- Place on magnetic stand to separate beads from supernatant 5min.
- Carefully transfer all supernatant to a new tube (contains all fragments <370bp!)

**Second bead selection to remove small fragments**

- Add 0.16 volumes (of the original volume, e.g., 16μl if original volume was 100μl) to the supernatant, mix well and incubate for 5-10 minutes at RT
- Put the plate on a magnetic stand and remove the supernatant after the solution cleared up (5 min).
- Wash the beads 2x with 200μl freshly prepared 80% EtOH while still on the magnetic stand, incubate for 1 min and carefully remove and discard the supernatant.
- Still on the magnetic stand, leave the plate to dry (with open cap) at RT for 5-10min. DO NOT OVERDRY! (lower DNA recovery rate)
- Remove the tube from the magnetic stand. Elute in 40μl (0.1X TE or ddH_2_O). Mix well by pipetting up and down and incubate for 2 minutes at RT.
- Put the tube back on the magnetic stand for 3 minutes. Transfer the supernatant to a clear tube.
- Pool both supernatants into a single tube.

**Third bead selection to remove adapter**

- Add 1.2 volumes (of the original volume, here the eluted one, approximately 80μl) to the cleaned-up DNA, mix well and incubate for 5-10 minutes at RT
- Put the plate on a magnetic stand and remove the supernatant after the solution cleared up (5 min). DO NOT DISTURB THE BEADS! (contain DNA targets)
- Wash the beads 2x with 200μl freshly prepared 80% EtOH while still on the magnetic stand, incubate for 1 min and carefully remove and discard the supernatant.
- Still on the magnetic stand, leave the plate to dry (with open cap) at RT for 5-10min. DO NOT OVERDRY! (lower DNA recovery rate)
- Remove the tube from the magnetic stand. Elute in 28μl (0.1X TE or ddH_2_O). Mix well by pipetting up and down and incubate for 2 minutes at RT.
- Put the tube back on the magnetic stand for 3 minutes. Transfer the supernatant to a clear tube.
- 1μl is kept to run on the Tapestation.

#### PCR

Set the reaction up on ice and quickly transfer the reaction to the preheated (98°C) thermocycler. The Phusion MasterMix is added last in order to prevent any primer degradation

**30μl reaction (10X)**

1.5μl primer 1 (10μM)

1.5μl primer 2_index (10μM)

0.9μl DMSO

15μl Phusion Mastermix 2X, NEB

2.4μl size selected DNA and 8.7μl ddH_2_O for 5X diluted PCR

**10 PCR cycles**

Initial Denat. 98°C 30s.

Denaturation 98°C 10s.

Annealing 65°C 30s 10X

Extension 72°C 30s

Keep @ 4°C ∞

#### Final MinElute Cleanup

Follow the manufacturer’s instructions, elute twice with 12μl -> will result in a total of 24 μl eluate. Use only a single column. Due to the large volume the PB/sample mixture will have to be loaded and centrifuged multiple times.

Run 1μl of the size selected and PCR product on the Tapestation (together with the size selected pre-PCR product).

#### Elchrom Size Selection

Prepare in advance:

- autoclaved 1X TAE buffer (at least 2 litres, *i.e.*, mix 40 ml 50X TAE with 1960 ml MiliQ water)

**Buffer refilling**

- Plug in water tubes and pump cables. Fill the tank with ddH_2_O. Start water pump and ensure that no water leaks into the running buffer.
- Fill gel tray halfway up, start the pump (this results in fewer air bubbles). With the pump running, add remaining buffer (up to 2000 ml).

**Preheating**

- Set temperature to 55°C. Turn on heater (black switch), water pump (green switch), and buffer pump (red switch and turning knob). Close lid.
- Turn on power unit (120 V, max. amperage).
- For the final 15-20 min, unpack gel and lay it onto the catamaran to preheat. Close lid.

**Sample preparation**

- Mix 5μl Elchrom loading buffer with 20μl library (load max 10μg of DNA per well to avoid overloading!).
- Prepare 0.3μl M3 marker with 1.5μl loading buffer and 3μl MiliQ water (3X).
- Take tips, pipettes, samples, tissues, and Kimwipes to the gel work bench.

**Gel loading (within 2-5 min)**

- When buffer is preheated and everything is ready, turn-off power unit.
- Turn buffer pump off (set to off, not power button), as well as the heater and water pump.
- Open lid and clean it with Kimwipes. Immerse gel in the gel tray, fix with catamaran.
- Remove any bubbles from the wells. Ensure gel is in the correct position.
- Load 12.5μl or 25μl of each library onto the gel (depending on gel).
- Load 5μl M3 Marker onto the gel.
- Close lid turn on power unit. Set to 120 V, max. Amperage, and estimated running time.
- Turn on water pump and heater (but keep the buffer pump off!).
- Start the run as soon as possible. Manually start the buffer pump after ~5 min.

**Staining**

- Turn off power source.
- Remove gel and use nylon string (6X at least!) to get rid of the back plate.
- Fill staining tray with 50 ml of MiliQ water, 336μl 50X TAE and 10μl SYBR gold. Slide the gel gently into the tray and wrap tray in aluminium foil.
- Stain on the shaker for 30-45 min.
- Empty staining tray without removing the gel and fill with 99 ml MiliQ water and 1 ml Destaining solution.
- Destain on the shaker for 30 min.
- Remove destaining solution.

**Size selection**

- Slide the gel on the DarkReader. Turn on and take a picture. Use a long-bladed knife and cut out the desired size range.

**Electro elution**

- Prepare as many dialysis membranes as needed (approximately 3 cm longer than the gel slice) and a few extra in various sizes. Wash the membranes in 1X TAE and store them in a basin with TAE until use.
- Close one end of membrane with a clip and fill membrane with 1ml of 1X TAE.
- Put the gel piece into the membrane. Make sure the gel piece is originated in previous running direction. Close membrane with a second clip. Avoid air bubbles.
- Place gel packages in the Elchrom gel chamber. Packages should be well covered with 1X TAE buffer but not floating.
- Run overnight at 20°C (change water), set Voltage to 90V.
- Reverse polarity for 1 min to detach DNA from the membrane.
- Clean-up with the MinElute PCR purification kit. On a glass plate, pipette out the TAE buffer and dispense into a Falcon tube containing 5ml of PB buffer. Rinse the membrane with buffer to maximise recovery rate.
- Apply 750μl of the mixture on a column at a time.

Elute in 2X 12μl EB buffer each, use Lo-bind tubes.

#### Library QC

- Qubit

- dsDNA HS kit according to instructions

- Tapestation

HS D1000 tape according to instructions

- qPCR

The qPCR is performed with the KAPA library quantification kit

The dilution of the library (1:10,000) is performed in ddH_2_O. Make three independent dilutions and pipet each dilution three times on the plate.

| Stage | Temperature | Time | Reads |
| --- | --- | --- | --- |
| Holding | 95°C | 5:00 min |  |
| Cycling | 95°C  60°C | 00:30 min  00:45 min | X |
| Melt curve | 95°C  60°C (ramp)  95°C  60°C | 0:15 min  1°C / 1:00 min  0:30 min  0:15 min | X |

Calculate the library concentration in nM using all the above obtained data. It should give you a similar range:

Qubit:

$concentration in nM=\frac{(concentration in ng/ul)}{(660g/mol*average library size bp)}$ * 10^6

Dilute the libraries to 4 or 2nM using 10nM Tris (pH 8.0) or based on the sequencing facility’s instruction

## ddRAD including degenerate bases for PCR duplicate detection (adapted from Tin et al 2014)

MseI motif

5‘ ... T TAA ... 3‘

3‘ ... AAT T ... 5‘

EcoRI motif

5‘ ... G AATTC ... 3‘

3‘ ... CTTAA G ... 35

**Oligos; Adapters; Digested genomic DNA (EcoRI and MseI)**

ACACTCTTTCCCTACACGACGCTCTTCCGATCTTCGAT AATTCNNNT TAGATCGGAAGAGCACACGTCTGAACTCCAGTCAC

|||||||||||||||||||||||||||||||||||||| |||| |||||||||||||||||||||||||||||||||

TGTGAGAAAGGGATGTGCTGCGAGAAGGCTAGAAGCTATTAA GNNNAAT CTAGCCTTCTCGTGTGCAGACTTGAGGTCAGTGCGGTTANNNNTAGAGCATACGGCAGAAGACGAAC

AATGATACGGCGACCACCGAGATCTACACTCTTTCCCTACACGACG 3’--> <--3’ AGCATACGGCAGAAGACGAAC

TruSeq P5/P7 read 1/2 TCTACACTCTTTCCCTACACGACGCTCTTCCGATCT GATCGGAAGAGCACACGTCTGAACTCCAGTCAC

**Final sequencing library (EcoRI from Widmer, MseI from Tin)**

TTACTATGCCGCTGGTGGCTCTAGATGTGAGAAAGGGATGTGCTGCGAGAAGGCTAGAAGCTATTAANNNTTACTAGCCTTCTCGTGTGCAGACTTGAGGTCAGTGXXXXXXNNNNTAGAGCATACGGCAGAAGACGAAC

||||||||||||||||||||||||||||||||||||||||||||||||||||||||||||||||||||||||||||||||||||||||||||||||||||||||||||||||||||||||||||||||||||||||||||

AATGATACGGCGACCACCGAGATCTACACTCTTTCCCTACACGACGCTCTTCCGATCTTCGATAATTNNNAATGATCGGAAGAGCACACGTCTGAACTCCAGTCACXXXXXXNNNNATCTCGTATGCCGTCTTCTGCTTG

**DNA sequence legend**

Sequencing Primer reads (PCR 1 Primer, PCR 2 Primer)

Primer (P5 and P7; flowcell annealing)

Restriction site

Barcode (1 of 48)

Index (1 of 12)

Degenerate base region

Genomic DNA
